# His Start-up Wants to Mimic Human Breast Milk Using Plants

**DOI:** 10.1021/acscentsci.6c01284

**Published:** 2026-08-06

**Authors:** Kristel Tjandra

## Abstract

Patrick Shih cofounded Totality Biosciences to make human milk oligosaccharides for more-nutritious baby formula

## Graphical abstract

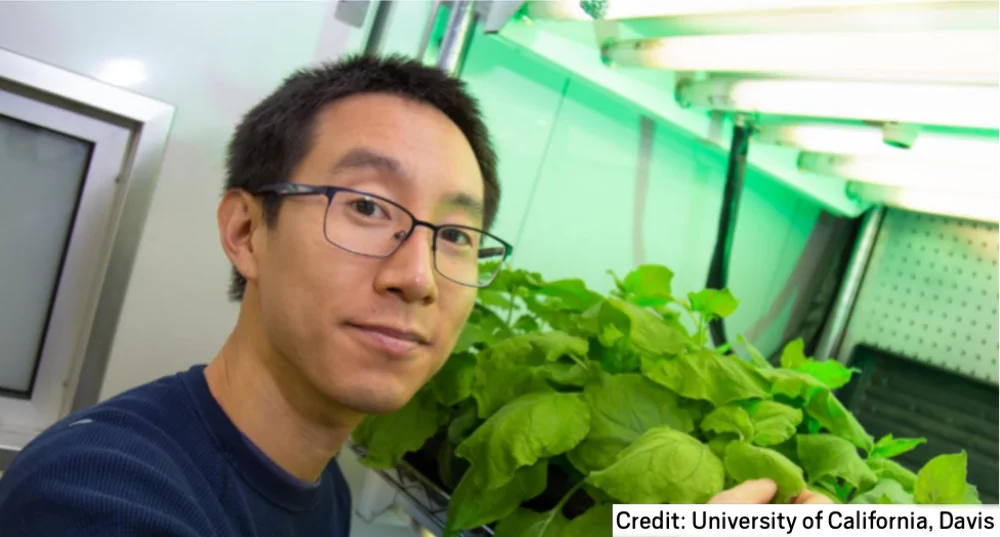

Breast milk is what sustains most babies in the first 6 months of their lives. Although parents have many options when choosing infant formula for their little ones, the composition of these products is much simpler than that of human breast milk. Aside from the myriad hormones and antibodies that human milk contains, it also carries hundreds of unique oligosaccharides—linear and branched chains of sugars—that have antimicrobial and anti-inflammatory properties that are beneficial for the infant’s health.
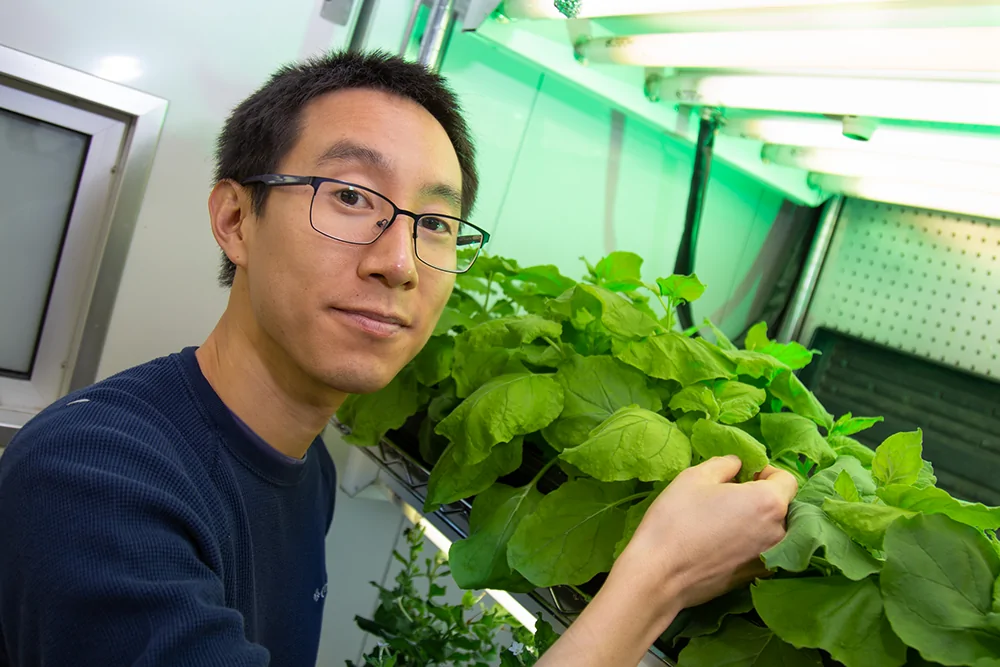


The US Department of Health and Human Services and Food and Drug Administration are currently working on Operation Stork Speed, an initiative to boost the nutritional quality of infant formula. One of the goals of this initiative is to make infant formulas more like human milk.

So far, fewer than 10 of the hundreds of known human milk oligosaccharides (HMOs) have been synthesized on a commercial scale and added to infant formulas. Often, only high-end formulas contain multiple HMOs.

Plant biologist Patrick Shih of the University of California, Berkeley, is determined to change that. Shih and his colleague Leila Strickland started Totality Biosciences, a start-up in the San Francisco Bay Area with a mission to produce all the HMOs found in human breast milk using a food crop.

Kristel Tjandra talked with Shih about the chemistry that drives this new venture.

## Take me back to the origin of your start-up.

My group has been engineering plant metabolism for a long time. Several years ago, we took a step back to figure out what would be an interesting class of compounds that plants are uniquely positioned to produce in large quantities. And arguably, sugar was the answer.

When we started this project, my lab was at UC Davis, where some of the best human milk oligosaccharide researchers in the world are. They suggested that I should think about HMOs.

I’d never heard of this class of compounds, but I did remember seeing them. I’ve had three kids, and I fed them formula. One night, sleep deprived, I was looking at the formula containers and realized, “Oh! They contain these HMOs.” And so I thought, “Yeah! We should give this a shot. Let’s try to engineer these into plants.”

So we did. And about 2 years ago, we put out a paper in *Nature Food* where, for the first time, we demonstrated we could produce HMOs—a wide variety of them—in plants.

## How wide a variety are we talking?

I will say, with a caveat, that it is a relatively small fraction of the total amount of, say, over 200 HMOs that you could find in human milk, but it was a good number of unique molecules any organism has ever produced.

What’s important to note here is that HMOs are bifurcating carbohydrates, meaning they are all made up of the same handful of building blocks. So imagine that you have a bunch of Lego bricks, and you can take the same Lego bricks but put them or assemble them in different ways to get different structures. For HMOs, there’s actually only 12 glycosidic linkages—Lego bricks—and you can use these linkages in different ways to get that full diversity of 200 HMO structures.

That is what we’ve been moving toward. In that initial *Nature Food* paper, we showed that we could reconstitute 7 of the 12, and by doing so, we were starting to chip away at the 200 structures. We’ve been able to make more than a dozen so far. But in theory, if we can make all 12 of those glycosidic linkages, we should be able to make all possible HMOs. That is the big North Star.

## You told me that this synthesis is all happening in a plant. What is the process like?

Back in the ’80s, plant scientists realized that *Agrobacterium tumefaciens*—a plant pathogen—could transfer a bit of their bacterial DNA from their plasmid into a plant’s nuclear genome and effectively transform it. That plant was essentially the first transgenic plant and what enabled agricultural biotechnology.

The plant we used is *Nicotiana benthamiana—*we just call it “Benthi.” It is like a cousin of tobacco that has emerged as a great model system in plant biology. So what’s cool is that you can take a syringe full of *Agrobacterium* that carries one of our engineered plasmids and literally squirt this bacterial solution onto Benthi’s leaf. And then many or all the plant cells in the leaf will transiently express whatever gene was in the *Agrobacterium*.

## And that worked all the time?

Well, pretty much whenever we engineer a pathway into a plant, it never works the first shot. It is a process that can take upward of 3 to 9 months. But we were shocked that Benthi was immediately very happily making HMOs. So within 2 to 4 days of taking in the engineered plasmid, the plant was transiently expressing the genes we wanted in its leaf.

This is the first time anyone’s engineered HMOs in a plant. And not only did we make one, two, or three of them, we made dozens of HMOs at the same time in a plant leaf, which was great.

## How does this process compare with using bioreactors?

When you make a de novo compound in, say, *Escherichia coli*, you are essentially making an HMO from sugar that is being fed into the bioreactor. What’s cool about photosynthetic organisms like plants is that you do not even have to feed them anything—well, other than sunlight, water, and fertilizer. It is the carbon dioxide that is actually being photosynthetically fixed and ultimately converted into the carbon atoms in the HMO.

## What about the yield? How does it match up?

In theory, we should be able to make much more product in an agricultural setup because the amount of inputs is intrinsically lower, and the area that you have to produce this compound is dramatically larger compared with a bioreactor.

Plus, we cannot choose one method over the other. There are no [biochemical facilities] that can make these HMOs.

In our paper, we did a techno-economic analysis to show that we could make some of the more complex HMOs in plants in large amounts and at reasonable prices—something that would’ve been extremely expensive to make in a bioreactor. That is where plants really shine.

## What are you most excited about in this whole development?

What I get excited about is how you can rethink what you can do with agriculture if you have the ability to engineer it.

Imagine a world where you could just make all the components—a huge number of components of human milk, not just the oligosaccharides but the many proteins and lipids that are really important, and what make human milk so special—in, say, a soybean plant and then turn those soybeans into milk. I know this is like a sci-fi idea, but that is what I get excited about.

*Kristel Tjandra is a freelance contributor to* Chemical & Engineering News *, the independent news publication of the American Chemical Society. Center Stage interviews are edited for length and clarity.*

